# Improving access and management of skin tumours: Over a decade of teledermoscopy in northern Sweden

**DOI:** 10.1177/1357633X261426680

**Published:** 2026-04-09

**Authors:** Virginia Zazo, Antonia Lindfors, Gabriel Michaëlsson, Zinaida Bucharbajeva, Senada Hajdarevic, Maja Af Klinteberg, Nirina Andersson

**Affiliations:** 1 Department of Public Health and Clinical Medicine, Dermatology and Venereology, 174459 Umeå University, Umeå, Sweden; 2 Department of Nursing, 8075 Umeå University, Umeå, Sweden

**Keywords:** Teledermoscopy, telemedicine, skin cancer diagnosis, melanoma, dermoscopy

## Abstract

**Introduction:**

Teledermoscopy (TD), introduced in Västerbotten, northern Sweden in 2014, enables dermatological consultation in primary care via high-resolution images. As skin cancer incidence increases in Sweden, TD offers potential for diagnostic efficiency and resource optimisation. Despite increasing referrals, TD remains unevaluated. This study examines TD's impact on equitable access to dermatological assessment for skin tumours in this region.

**Methods:**

This descriptive study analysed 67,137 TD referrals submitted between 2014 and 2024, excluding 2,384 due to incomplete data. Variables included age, diagnosis (benign vs malignant), and referral origin (public/private; urban/rural). A survey examined TD routines across primary care centres (PCCs), staff roles, training, and frequency of internal TD discussions.

**Results:**

Over the 11-year period, mean age increased from 50 (2014) to 61 years (2024) (*P* < 0.001). The proportion of benign referrals decreased from 80% to 69% (*P* < 0.001). Private PCCs referred 78% benign lesions versus 75% from public PCCs (*P* < 0.001); urban PCCs referred 77% versus 73% PCCs in remote areas (*P* < 0.001). Assessment by a nurse was associated with higher benign referral rates (odds ratio (OR) 1.132, 95% confidence interval (CI) 1.052–1.219), whereas internal TD discussions (OR 0.714, 95% CI 0.669–0.763) and lack of dermoscopy training (OR 0.893, 95% CI 0.882–0.971) were associated with lower benign referral rates.

**Discussion:**

TD has enhanced access to dermatological evaluation for suspected skin tumours in Västerbotten. The increasing age of referred patients and higher proportion of tumour diagnoses imply improved targeting of high-risk groups. Local routines influenced referral quality, underscoring the need for structured TD implementation.

## Introduction

Approximately 5,000 patients are diagnosed with cutaneous malignant melanoma (CMM) each year in Sweden.^1^ Diagnosing CMM is time-consuming and often requires evaluation by experienced general practitioners (GPs) or dermatologists, which can contribute to delayed diagnoses.^2^ Early diagnosis is crucial for improving prognosis, yet CMM still accounts for 500 deaths annually in Sweden.^3^ One of the key prognostic factors is time to diagnosis, making it essential to reduce diagnostic delays.^4^

Diagnostic uncertainty has led to unnecessary excisions of benign lesions, straining healthcare resources.^5^ This underscores the importance of improving diagnostic accuracy.

To address these issues, the Swedish Association of Local Authorities and Regions, together with the National Board of Health and Welfare, launched the Cancer Patient Pathways initiative (SVF) to shorten the time between diagnosis and treatment. CMM was among the first cancers included in 2016.^6^

Dermoscopy, a non-invasive method using a dermoscope to examine skin lesions, is widely used by GPs and dermatologists in face-to-face consultations. It offers high diagnostic accuracy and is considered the gold standard for evaluating suspicious lesions.^7^ However, its effectiveness depends on the examiner's experience.^8^

Teledermoscopy (TD) is a referral method enabling remote consultations using dermoscopic images. In Västerbotten County, northern Sweden, TD is used in primary care centres (PCCs), where GPs or trained nurses capture dermoscopic and macroscopic images of one to three lesions using a smartphone with a dermoscope attachment. These, along with a brief clinical history, are sent via an electronic health record to a dermatologist, who assesses the case and returns a management plan. In urgent cases or suspected melanoma, the standardised cancer care pathway (SVF) is activated. SVF is a national Swedish initiative launched in 2016 to reduce diagnostic delays. In the context of TD, SVF is activated for suspected melanoma cases. While PCC procedures vary, certain TD routines (e.g. consultation structure, staff roles) are informed by regional guidelines developed in Västerbotten.

Two main types of TD exist: store-and-forward and real-time interactive. Store-and-forward, the method used in Västerbotten, involves capturing and later transmitting images for dermatologist review. Real-time interactive TD allows immediate video consultations between patient and dermatologist. While dermoscopy has long been the diagnostic gold standard for skin tumours, recent studies show that TD offers comparable diagnostic accuracy.^6,9^

Most TD studies have been conducted outside Sweden, either evaluating existing systems or within experimental frameworks. An Australian systematic review showed that TD reduced waiting times and improved diagnostic timelines while maintaining high patient satisfaction.^10^ Other studies report improved access to dermatology for underserved groups, such as rural residents and older adults.^11–14^ TD's diagnostic accuracy has remained high across studies, including a reported sensitivity of 98.9% in Västerbotten.^15–17^

Previous analyses found the number needed to treat for TD to be as low as 2.06–2.60, meaning one confirmed malignancy for every two to three excised lesions.^18,19^ This highlights TD's diagnostic efficiency and potential to optimise surgical resource use. From an economic standpoint, TD has demonstrated more cost-effectiveness than face-to-face consultations.^20^

Before TD implementation, patients in Västerbotten accessed specialist evaluations through various channels, including an open-access mole clinic at Umeå University Hospital. However, this mainly attracted younger patients, living within 50 km of the hospital, while those most at risk often were overlooked. Meanwhile, 75% of patients with superficial spreading or nodular melanoma were initially treated at PCCs, but with longer delays and more advanced disease than those treated at hospitals.^21^

TD was introduced in Västerbotten in 2014 and is now used by all PCCs except one. Referral volumes have since increased substantially, straining existing dermatology resources. Despite its widespread use, TD has not yet been formally evaluated in this region. Prior studies have focused on diagnostic accuracy, patient satisfaction, and variables such as sex, age, and time to assessment, often within distinct healthcare settings.^22–24^ With limited specialist dermatology capacity in rural Västerbotten, evaluating whether TD facilitates equitable access to specialist care is essential.

The present study aimed to evaluate whether TD facilitates equitable dermatological assessment of cutaneous tumours in Västerbotten. Specifically, we examined TD usage, characteristics of referred patients (age, sex, diagnosis, referral unit), and whether the dermatologist's preliminary diagnosis was associated with patient demographics or referral structure (urban vs rural location; public vs private PCC). We also explored how primary care routines affect referral patterns.

## Methods

### Subjects

In this descriptive study, all TD referrals sent by PCCs in Västerbotten to the Dermatology and Venereology Department at Umeå University Hospital between 2 May 2014 and 24 November 2024 were included. During this period, 67,136 cutaneous lesions were evaluated. Of these, 2,384 lesions were excluded due to missing referral unit data (*n* = 1,773), absent diagnostic information (*n* = 151), or irrelevant diagnoses (*n* = 460; ICD-10 Z codes, i.e. factors influencing health status and contact with health services, according to the WHO's ICD-10-SE classification), leaving 64,752 lesions for analysis.

Each TD referral included between one and three cutaneous lesions, and patients could appear multiple times in the dataset if they had been referred on more than one occasion during the study period. All TD evaluations were performed by dermatologists with specialised training in TD at the Dermatology and Venereology Department of Umeå University Hospital, some of whom contributed remotely, from locations outside the hospital setting.

For each lesion, the following variables were recorded: patient sex, age, diagnosis, date of referral, place of residence, and referring PCC. The PCCs were categorised based on their geographical proximity to the dermatology department, using a 50 km threshold to distinguish between urban (≤50 km) and rural (>50 km) settings. This cut-off was chosen to reflect practical travel constraints for patients and to ensure consistency with a previous local evaluation conducted before the implementation of TD. PCCs were also classified by management type (public vs private). The dataset comprised 481 distinct diagnostic codes, which were grouped into four main categories: CMM, non-melanoma skin cancer (NMSC), benign lesions, and suspected tumours (clinically suspicious tumours of undefined or indeterminate nature). For analytical clarity, these categories were further condensed into two overarching groups: tumours (including CMM, NMSC, and suspected tumours) and benign lesions.

### Surveys

In the autumn of 2024, a survey was distributed to all 40 PCCs in Västerbotten that were actively using TD (Supplemental file 1). Of these, 32 PCCs (80%) completed the survey. The purpose of the survey was to explore whether local implementation routines were associated with the types of skin lesions referred for TD evaluation. The survey covered various aspects of cutaneous lesion management, including organisational structure, pre-assessment procedures, and the roles of healthcare professionals involved in the referral process. Specific questions addressed which staff categories were responsible for patient assessment, lesion photography, and referral decisions. The survey also examined criteria used for TD referrals, the extent of staff training in TD, and the presence of workplace routines such as regular meetings or designated TD coordinators. Responses were collected from either the head of the PCC or the medically responsible physician. The survey data were subsequently linked to individual TD referrals, enabling an analysis of how local routines may influence referral patterns.

### Statistical analyses

Descriptive analyses were conducted using Microsoft Excel (Version 16.91), and statistical analyses were performed in SPSS (Version 28.0, IBM Corp.). One-way analysis of variance was used to analyse trends in mean patient age over time, while the Cochran–Armitage test for trend assessed changes in the proportion of benign lesions across the study period. The chi-squared test examined associations between dichotomous variables and lesion type, with statistical significance set at *P* < 0.05.

Univariate analyses were initially conducted to explore associations between each independent variable and the dependent variable, defined as lesion type (benign vs tumour). Independent variables included patient age, sex, year of referral, location and type of PCC, and local implementation routines – such as staff training in TD, the healthcare professional initiating the referral, and whether TD was regularly discussed during workplace meetings. Variables with a *P*-value <0.05 in the univariate analysis (age, sex, referral year, PCC type and location, and local TD routines (as detailed in Table 1)) were included in a multi-variable logistic regression model to control for potential confounding and to evaluate the independent effect of each factor on lesion type.

**Table 1.** Survey responses from 32 primary care centres regarding teledermoscopy (TD) practices, and their association with referrals for benign lesions.

Survey category	Response options	Most common response (*n*)	Univariable OR (95% CI)	Multi-variable OR (95% CI)
Consultation structure	Planned/drop-in/mixed	Planned (29/32)	Planned consultation **0.877 (0.830–0.926**)	**0.729** (**0.669–0.794)**
Pre-consultation questions asked	Yes/no	Yes (24/32)	Pre-consultation questions asked **1.064 (1.019–1.111)**	1.039 (0.976–1.106)
TD availability frequency	Daily/weekly/monthly/sporadic	Monthly (15/30)	TD weekly or more often 0.995 (0.956–1.035)	Excluded in multi-variable analysis
Average patients/week for lesion assessment	Numeric value	Median: 7 patients/week	Fewer than 7 patients per/week 0.980 (0.941–1.021)	Excluded in multi-variable analysis
Personnel performing clinical assessment	GP/nurse/other	GP (32/32)	Assessment by a nurse **1.077 (1.031–1.126**)	**1.132** (**1.052–1.219)**
Personnel taking TD photos	GP/nurse/other	GP (16/31)	Photo by GP 0.990 (0.953–1.029)	Excluded in multi-variable analysis
Are all patients referred via TD?	Yes/almost/far from all	Almost (17/32)	Referring far from all cutaneous lesions **0.945 (0.910–0.982)**	1.032 (0.977–1.091)
TD referral criteria	Free text	Suspected tumour (16/28)	Referring not only suspected tumours 1.024 (0.969–1.083)	Excluded in multi-variable analysis
Intro TD training for new staff	Yes/sometimes/no	Sometimes (17/29)	TD training for all new staff **1.078 (1.036–1.122)**	**0.908** (**0.836–0.987)**
Participation in RCC e-course	Yes/some personnel/no	Some personnel (18/31)	Lack of RCC e-course **1.128 (1.050–1.211**)	**1.172** (**1.009–1.360)**
TD discussed at staff meetings	Yes/no	Yes (20/32)	TD discussed at staff meetings **0.818 (0.787–0.850**)	**0.714** (**0.669–0.763)**
TD coordinator assigned	Yes/no	No (24/31)	TD coordinator **0.951 (0.909–0.995)**	**1.249** (**1.145–1.362)**
GP trained in dermoscopy	Yes/no	Yes (18/31)	Lack of dermoscopy training **0.811 (0.771–0.854**)	**0.893** (**0.882–0.971)**

OR: odds ratio; CI: confidence interval; RCC: regional cancer centre; GP: general practitioner.

Statistically significant associations are indicated in bold.

## Results

### Overall diagnostic distribution

Of the 64,752 cutaneous lesions assessed via TD, 114 (0.2%) received a preliminary diagnosis of CMM, 8,429 (13.0%) as NMSC, 7,482 (11.6%) as suspicious tumours, and 48,727 (75.2%) as benign lesions. These diagnoses were based solely on TD and not histopathologically confirmed. The mean age of the patients increased steadily, from 50 years in 2014 to 61 years in 2024 (*P* < 0.001) (Figure 1). The median age also increased from 51 years in 2014 to 66 years in 2024. The median age of patients varied by diagnostic category: CMM – 66 years, NMSC – 72 years, benign lesions – 56 years, and suspected tumours – 66 years.

**Figure 1.**
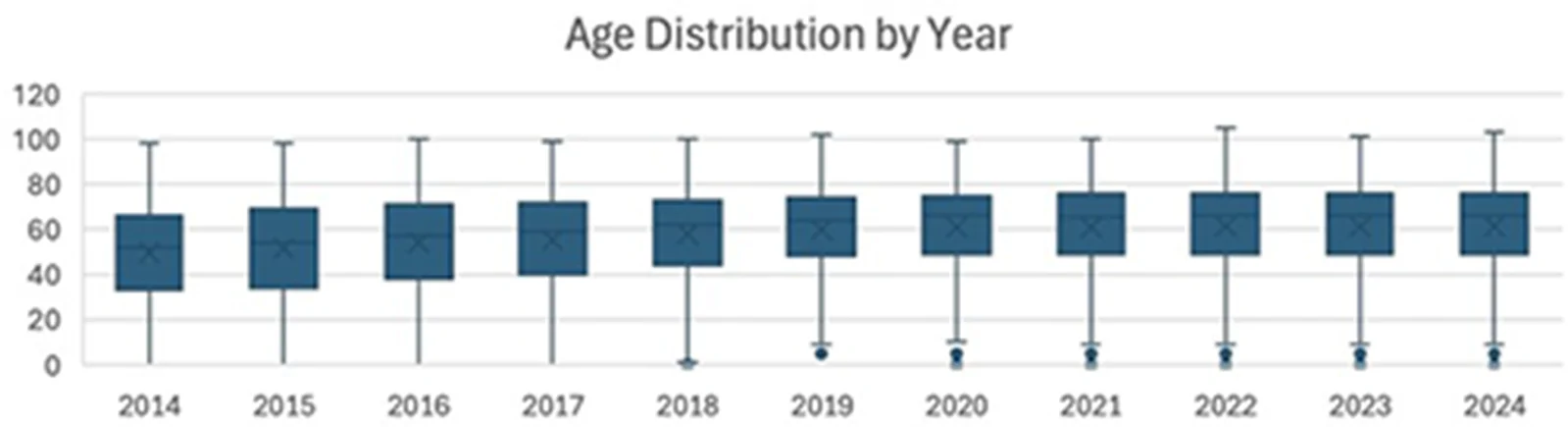
Boxplot showing patient age distribution by year. The mean age increased from 50 to 61 years, and this trend was statistically significant (*P* < 0.001; one-way analysis of variance).

### Trends over time

When analysing the types of skin lesions referred through TD over time, a steady annual increase was observed across all diagnostic categories, except for CMM, which remained relatively stable. Despite the overall increase in referral volume, a Cochran–Armitage test for trend demonstrated that the proportion of benign lesions decreased significantly relative to tumour-related assessments over time (*P* < 0.001) (Figure 2). The sex distribution remained stable throughout the study period, with approximately 60% of referrals concerning women and 40% men. A significantly higher proportion of men received a tumour diagnosis than women (27% vs 23%, *P* < 0.001).

**Figure 2.**
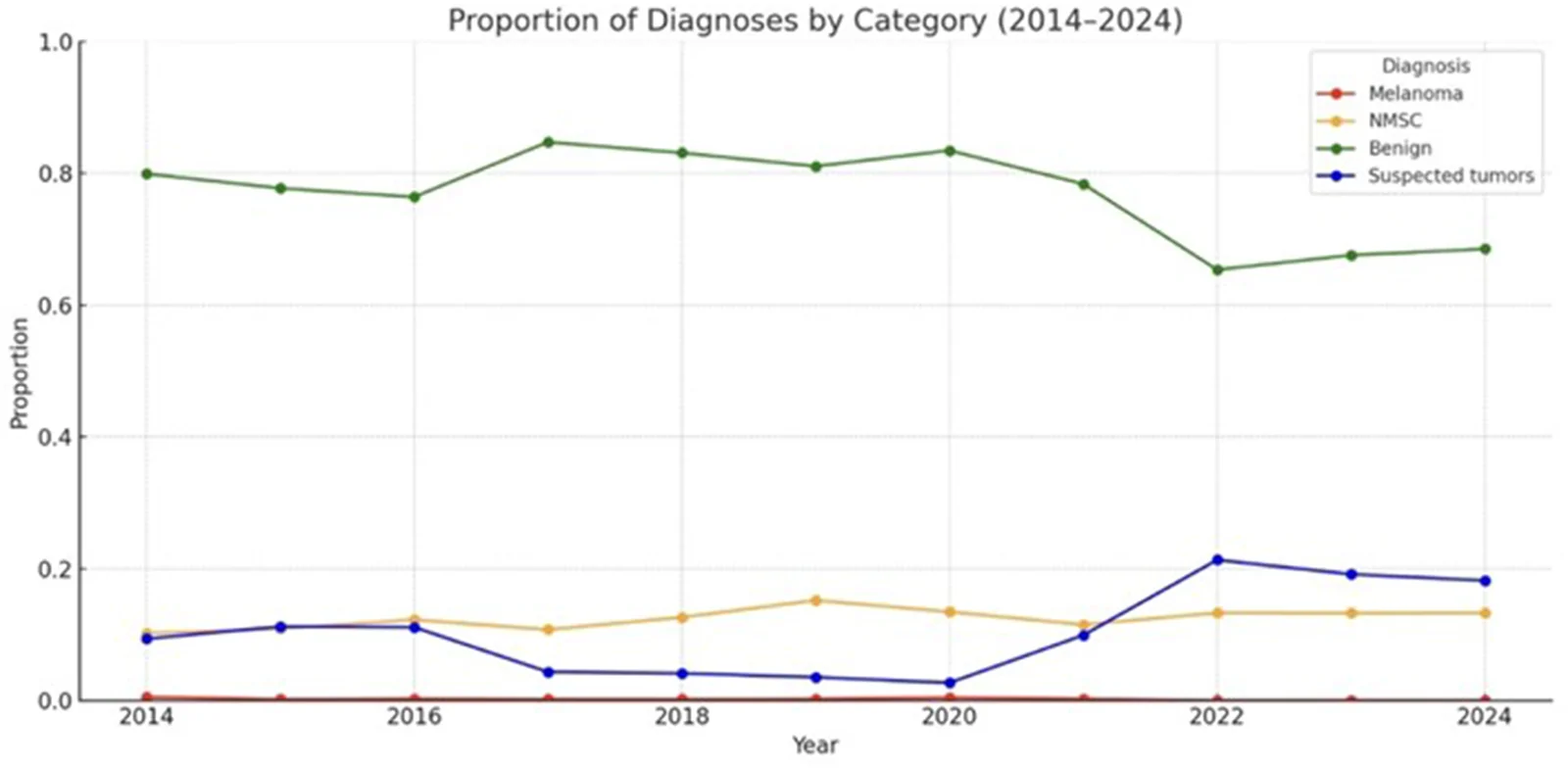
Annual proportion of each diagnostic category among teledermoscopy referrals in Västerbotten (2014–2024). The proportion of benign lesions declined significantly from 78.7% in 2014 to 65.5% in 2024 (*P* < 0.001; Cochran–Armitage test for trend).

### Geographic and organisational referral patterns

Rural PCCs submitted 30,833 referrals, while urban PCCs near the dermatology department accounted for 33,919. Rural PCCs were significantly less likely to refer benign lesions (73% vs 77%, *P* < 0.001). Additionally, private PCCs contributed 10,846 referrals compared 53,906 from public PCCs. Private PCCs were more inclined to refer benign lesions than public ones (78% vs 75%, *P* < 0.001).

### Impact of primary care practices on referral quality

Univariate analysis demonstrated that specific procedures at PCCs significantly influenced the proportion of benign lesions referred via TD. Several associations remained statistically significant after adjusting for potential confounders in a multi-variable logistic regression model (Table 1). Assessment by a nurse was associated with a higher proportion of referrals assessed as benign (odds ratio (OR) 1.132, 95% confidence interval (CI) 1.052–1.219). A planned consultation structure was linked to lower rates of referrals assessed as benign (OR 0.729, 95% CI 0.669–0.794). Lack of participation in a free, nationwide, self-paced distance education programme developed by the Regional Cancer Centres in Collaboration (RCC) – designed to equip primary care staff with essential skills in dermoscopic imaging, risk assessment, and referral of suspected skin cancer – was significantly associated with a higher proportion of referrals later assessed as benign (OR 1.172, 95% CI 1.009–1.360). In contrast, GPs without formal dermoscopy training (OR 0.893, 95% CI 0.882–0.971), as well as PCCs where workplace discussions on TD were conducted (OR 0.714, 95% CI 0.669–0.763), had lower odds of submitting referrals subsequently classified as benign.

## Discussion

This study provides a detailed account of how TD has been implemented and utilised across Västerbotten County over more than a decade. While comparisons with historical data are limited due to the lack of consistent pre-implementation benchmarks, the patterns observed offer compelling insights into the reach and functionality of the TD system within current practice.

Before the implementation of TD, the management of suspected skin lesions in Västerbotten included both a referral-free, walk-in clinic at the dermatology department in Umeå and traditional referrals from PCCs. In our previous study from 2014,^21^ 75% of patients diagnosed with superficial spreading or nodular melanoma were initially managed by PCCs. These patients had significantly thicker tumours and longer times to histopathological registration compared to those treated in hospital-based dermatology settings. Additionally, unpublished data from the walk-in clinic indicated that the majority of patients assessed there were younger and often lived near the dermatology department, highlighting unequal access to specialist care. In contrast, TD referrals now represent a broader demographic distribution, including older patients and those residing in remote areas. This is reflected in the rising median age of referred patients, which now aligns more closely with national melanoma statistics, and in the increased proportion of referrals assessed as malignant. These shifts suggest more targeted triage and improved resource allocation.

Moreover, TD appears to have helped equalise dermatological access between urban and rural regions. Despite a similar population base, referrals from remote PCCs now match those from urban areas, supporting the conclusion that TD has closed a substantial geographical gap. This observation is consistent with previous studies highlighting the benefits of TD in underserved areas and rural populations.^13,14^ Interestingly, remote PCCs submitted proportionally fewer benign referrals than those located near the dermatology department. This could reflect more conservative and experience-based referral practices in areas with historically limited access to specialist care. A similar trend was identified in an Australian study, where remote GPs demonstrated higher rates of malignant lesion detection.^25^ Although the proportion of benign referrals was significantly higher in private PCCs (78%) than in public ones (75%, *P* < 0.001), the absolute difference is relatively small and should be interpreted with caution in terms of clinical impact. However, given the overall high volume of incoming referrals, even minor proportional differences may still have implications for the total referral burden and resource allocation. Such differences, although small, are particularly noticeable in public PCCs as they account for the majority of TD referrals.

Regarding sex distribution, our findings confirm the pattern previously seen in Swedish data^26–29^: women accounted for about 60% of TD referrals, although men generally have a higher incidence of CMM. Within the tumour group, however, men were more likely to be diagnosed with malignant lesions, suggesting that triage accuracy was maintained despite the sex imbalance in referral volume. Although histopathological confirmation was not available, this pattern aligns with the established epidemiological trends.

A particularly novel contribution of this study is the exploration of how specific routines at the primary care level influence referral quality. Factors such as whether referrals were initiated by a physician or nurse, whether nearly all skin lesions were referred, the presence of structured TD discussions at the workplace, and staff training participation were all significantly associated with the likelihood of benign referrals. Notably, PCCs where staff had not taken part in online TD education provided by the Confederation of Regional Cancer Centres (RCC) were more likely to refer benign lesions. On the contrary, physicians without formal dermoscopy training also submitted fewer benign referrals – a finding that may appear counter-intuitive, but could reflect greater diagnostic caution among trained clinicians, particularly in ambiguous presentations such as amelanotic melanoma. The Dunning–Kruger effect may offer partial explanatory insight; whereby increased knowledge brings greater recognition of diagnostic complexity.^30–32^ However, as PCC routines were surveyed in 2024 whereas referrals were recorded over the prior decade, routine changes over time may limit the interpretation of these associations.

This study benefits from a robust dataset, comprising over 64,000 lesions assessed over a 10.5-year period. This large sample size, along with high PCC participation, ensures representativeness and statistical power. The use of recent data minimises bias from demographic shifts and supports the study's validity within its setting. We acknowledge that some lesions may have been referred more than once during the study period; however, anonymisation of data and the lack of lesion-specific identifiers prevented adjustment for repeated referrals. Prior work from our group found that only 3 of 135 melanomas had undergone a previous TD evaluation of the same lesion within 2 years, indicating a low frequency of repeated referrals.^17^

The diagnostic classifications in this study are based on clinical assessments via TD, as no histopathological confirmation was available. This limits the ability to fully validate diagnostic accuracy. In the context of an observational design, it is not possible to establish causal relationships. Although randomised controlled trials would provide stronger evidence, such designs are neither feasible nor appropriate for evaluating an already widely implemented referral system. Still, the consistency of observed trends with national cancer statistics suggests strong external validity.^1^

The increased use of TD has led to a higher total number of assessments over time. Improved diagnostic precision is suggested by the proportional decrease in benign lesions and the concurrent increase in suspected tumours. The consistently low number of referrals diagnosed as melanoma throughout the study period suggests that TD primarily serves as a triage tool rather than a definitive diagnostic method for melanoma. Notably, between 2021 and 2022, melanoma referrals declined markedly, while diagnoses categorised as suspected tumours increased. Although no causal link can be confirmed, the decrease in melanoma diagnoses and increase in suspected tumours may reflect greater diagnostic caution, possibly related to changes in SVF routines, more complex or ambiguous lesions, and pandemic-related care delays.

## Conclusions

This study provides a comprehensive overview of TD implementation in Västerbotten, Sweden, over an 11-year period. The findings demonstrate evolving and increasingly structured referral patterns, improved alignment with high-risk patient groups, and reduced geographical disparities in access to dermatological expertise.

TD shows strong potential as a scalable and efficient tool for early skin cancer assessment in diverse healthcare settings. While this study highlights how local routines influence referral quality, the integration of artificial intelligence may help standardise assessments and reduce variability in referral practices. Future research should further evaluate diagnostic accuracy through histopathological correlation, assess patient-centred outcomes, and explore the long-term integration and cost-effectiveness of TD in routine clinical practice.

## Abbreviations

CMM: cutaneous malignant melanoma
GP: general practitioner
NMSC: non-melanoma skin cancer
PCC: primary care centre
RCC: regional cancer centre
TD: teledermoscopy

## Supplemental Material

sj-docx-1-jtt-10.1177_1357633X261426680 - Supplemental material for Improving access and management of skin tumours: Over a decade of teledermoscopy in northern Sweden Supplemental material, sj-docx-1-jtt-10.1177_1357633X261426680 for Improving access and management of skin tumours: Over a decade of teledermoscopy in northern Sweden by Virginia Zazo, Antonia Lindfors, Gabriel Michaëlsson, Zinaida Bucharbajeva, Senada Hajdarevic, Maja Af Klinteberg and Nirina Andersson in Journal of Telemedicine and Telecare

## Data Availability

The datasets generated and/or analysed during the current study are not publicly available due to ethical and privacy considerations, but are available from the corresponding author on reasonable request and subject to approval by the Swedish Ethical Review Authority.
