# Supplementary material for: Improving access and management of skin tumours: Over a decade of teledermoscopy in northern Sweden
Source: J Telemed Telecare. 2026 Apr 9;32(9):766–72. doi: 10.1177/1357633X261426680 (PMC13669448; doi:10.1177/1357633X261426680)
Supplement: Supplementary file 1 [file sj-docx-1-jtt-10.1177_1357633X261426680.docx]

# Supplementary File 1: Survey Summary

As part of this study, a survey was sent to all primary care centres (PCCs) in Västerbotten that use teledermoscopy (TD).

The purpose was to understand local implementation routines and their potential impact on referral quality.

The survey was distributed in autumn 2024 and completed by 32 of 40 PCCs (80% response rate).

Each response was submitted by either the head of the PCC or the medically responsible physician.

The survey included the following questions:

1. How is the cutaneous lesion assessment organized at the PCC?
2. Does the healthcare worker ask any specific questions to the patient before booking cutaneous lesion assessment?
3. How often is TD used at your center?
4. On average, how many patients per week are assessed using TD at your center?
5. Which category of healthcare staff sees the patient for a cutaneous lesion assessment?
6. Who photographs the cutaneous lesion for TD?
7. Are all patients booked for cutaneous lesion assessment referred for TD?
8. Under what condition is a TD referral sent?
9. Do new employees get an introduction to TD?
10. Do the employees participate in the online training on TD for PCCs, available via the Confederation of Regional Cancer Centres in Sweden?
11. Is TD informed/discussed at the PCCs' workplace meetings?
12. Is there a dedicated person responsible for TD at the PCC?
13. Have any of the doctors at the PCC received training in dermoscopy?

Survey responses were compiled and linked to individual lesion-level TD referrals using unique PCC identifiers. This enabled an analysis of how local organisational practices influence referral patterns.
